# Physical Layer Security Enhancement in IRS-Assisted Interweave CIoV Networks: A Heterogeneous Multi-Agent Mamba RainbowDQN Method

**DOI:** 10.3390/s25206287

**Published:** 2025-10-10

**Authors:** Ruiquan Lin, Shengjie Xie, Wencheng Chen, Tao Xu

**Affiliations:** College of Electrical Engineering and Automation, Fuzhou University, Fuzhou 350108, China; 230127186@fzu.edu.cn (S.X.); 220110006@fzu.edu.cn (W.C.); 230127175@fzu.edu.cn (T.X.)

**Keywords:** CIoV, physical layer security, intelligent reflecting surface (IRS), resource allocation, dynamic spectrum access (DSA), multi-agent reinforcement learning

## Abstract

The Internet of Vehicles (IoV) relies on Vehicle-to-Everything (V2X) communications to enable cooperative perception among vehicles, infrastructures, and devices, where Vehicle-to-Infrastructure (V2I) links are crucial for reliable transmission. However, the openness of wireless channels exposes IoV to eavesdropping, threatening privacy and security. This paper investigates an Intelligent Reflecting Surface (IRS)-assisted interweave Cognitive IoV (CIoV) network to enhance physical layer security in V2I communications. A non-convex joint optimization problem involving spectrum allocation, transmit power for Vehicle Users (VUs), and IRS phase shifts is formulated. To address this challenge, a heterogeneous multi-agent (HMA) Mamba RainbowDQN algorithm is proposed, where homogeneous VUs and a heterogeneous secondary base station (SBS) act as distinct agents to simplify decision-making. Simulation results show that the proposed method significantly outperform benchmark schemes, achieving a 13.29% improvement in secrecy rate and a 54.2% reduction in secrecy outage probability (SOP). These results confirm the effectiveness of integrating IRS and deep reinforcement learning (DRL) for secure and efficient V2I communications in CIoV networks.

## 1. Introduction

Intelligent Transportation Systems (ITSs) [1], as an essential component of smart cities, are gradually evolving into an integrated framework that combines sensing, communication, and intelligent decision-making. Within this framework, the Internet of Vehicles (IoV) [2,3] plays a pivotal role by enabling large-scale, low-latency, and high-reliability information exchange, thereby supporting traffic safety, road management, and mobility services [4,5].

Among various communication modes in IoV, Vehicle to Vehicle (V2V) and Vehicle to Infrastructure (V2I) are two fundamental paradigms [6,7]. V2V communication relies on direct links between neighboring vehicles, enabling distributed information sharing. However, its coverage is limited and highly sensitive to vehicle speed and density [8]. In contrast, V2I communication leverages roadside units or base stations as relays, offering wider coverage and more stable connectivity. This makes V2I particularly suitable for critical applications such as traffic signal optimization, road safety alerts, and route planning. Consequently, ensuring the security and reliability of V2I communications under highly dynamic vehicular environments has become a key research challenge in IoV.

With the rapid expansion of IoV, wireless communication systems are facing increasing spectrum scarcity. Fixed spectrum allocation can no longer satisfy the bandwidth demands of massive vehicular access and dynamic traffic services, resulting in inefficient spectrum utilization. To address this issue, cognitive radio (CR) [9,10] has been introduced into IoV, resulting in the formation of the Cognitive Internet of Vehicles (CIoV) [11,12]. In a CR system, primary users (PUs) are licensed users with priority access to the spectrum, while secondary users (SUs) are unlicensed devices that opportunistically access idle spectrum. CR enables environmental sensing and dynamic spectrum access, allowing SUs to opportunistically utilize idle spectrum when it is not occupied by PUs, thereby significantly improving spectrum efficiency. Depending on the access mechanism, CR can operate in three typical modes: overlay, underlay, and interweave. Among them, the interweave mode has attracted particular attention, as it enables efficient spectrum sharing while guaranteeing the protection of PUs [13]. In this mode, SUs are permitted to access the channel only when spectrum holes are detected. This property makes interweave mode well-suited to the requirements of V2I communications in IoV. Compared with the distributed access in V2V, V2I communications under interweave mode, supported by infrastructure, can achieve larger coverage and more stable connectivity. However, the resource allocation must simultaneously address the challenges of spectrum dynamics and secure transmission, which significantly increases the complexity. This serves as the primary motivation of this study on resource allocation for V2I communications in an interweave CIoV network.

The openness and broadcast nature of the CIoV network make it highly vulnerable to physical layer attacks, particularly passive eavesdropping [14,15]. Eavesdroppers (Eves) can threaten legitimate transmissions merely by intercepting V2I signals without introducing active interference. To address this security concern, Intelligent Reflecting Surface (IRS) [16,17], an emerging reconfigurable environment technology, has recently been introduced into physical layer security communications [18,19]. By adaptively adjusting the phase shifts of its reflecting elements, IRS is capable of intelligently reconfiguring the wireless propagation environment. This allows it to enhance the strength of legitimate links while suppressing eavesdropping channels, thereby significantly improving system security with low power consumption and low hardware complexity.

## 2. Related Work

Recent studies have highlighted the effectiveness of IRS in vehicular communication systems. Yang et al. [20] proposed a weighted performance optimization algorithm using alternating optimization to jointly design IRS phase shifts and beamforming, mitigating resource conflicts among sensing, communication, and computation. Cao et al. [21] introduced an IRS-assisted framework based on statistical CSI, employing the JAPBNB algorithm for joint active and passive beamforming to maximize the sum-rate in multi-vehicle scenarios. Duan et al. [22] further developed an IRS-enhanced V2X framework that jointly optimizes mode selection, spectrum reuse, computation offloading, and beamforming, reducing latency and energy consumption in dynamic vehicular environments.

In CR networks, IRS technology has also received widespread attention. In [23], an IRS-assisted UAV communication framework was proposed, where the UAV trajectory, IRS beamforming, and power allocation are jointly optimized to maximize the SUs throughput under PUs interference constraints. Similarly, ref. [24] studied an IRS-aided MISO cognitive radio system, where the transmit and reflective beamforming are jointly optimized to maximize the SUs rate while satisfying PUs interference and IRS power constraints, demonstrating significant throughput improvements. Moreover, in [25], an IRS-assisted CR system with opportunistic spectrum sharing was considered, supporting both secondary transmission and spectrum sensing. A two-stage IRS phase shift optimization algorithm based on block coordinate descent (BCD) was proposed to maximize the average secondary transmission rate.

Recent studies have shown that IRS effectively mitigated eavesdropping and enhanced physical layer security. In [26], IRSs in underlay cognitive radio networks using selection combining (SC) and maximal ratio combining (MRC) significantly reduced SOP and increased the probability of nonzero secrecy capacity (PNSC). A hybrid IRS with element on–off control and two-stage beamforming–amplification optimization was proposed in [27], improving PLS with lower power consumption. Additionally, ref. [28] optimized IRS reflection under uncertain eavesdropper locations, which enhanced PNSC and ergodic secrecy capacity while reducing SOP.

The aforementioned works mostly rely on conventional resource management and optimization techniques to tackle the inherently non-convex problems in IRS-assisted networks. However, such methods typically incur high computational complexity, require accurate mathematical modeling. In contrast, DRL is model-free and capable of learning near-optimal policies directly through interaction with complex and uncertain environments, which makes it particularly promising for IRS-assisted CIoV systems. Zhao et al. [29] applied DRL to adjust UAV trajectories and IRS phase shifts in real time, maximizing communication rate. Qi et al. [30] employed SAC-based DRL to jointly optimize IRS phase shifts and vehicular resource allocation, minimizing age of information and enhancing payload delivery. Dong et al. [31] used PPO-based DRL to optimize UAV trajectory, beamforming, and IRS phase shifts, improving the secrecy rate and reducing outage under eavesdropper CSI. Qin et al. [32] applied DDPG-based DRL to jointly optimize channel allocation, transmit power, and STAR-IRS coefficients in NOMA systems with dual eavesdroppers, achieving a robust sum secrecy rate. Ju et al. [33,34] recently explored AI-assisted physical layer security in vehicular networks. Specifically, ref. [33] proposed a NOMA-assisted secure offloading scheme for vehicular edge computing using cooperative jamming and asynchronous deep reinforcement learning to reduce energy consumption under latency constraints, while ref. [34] developed a deep recurrent reinforcement learning-based framework for mmWave vehicular networks, jointly optimizing beam allocation, relay/jammer selection, and transmit power to enhance secrecy capacity and energy efficiency. These studies collectively demonstrate the effectiveness of DRL for real-time joint optimization in IRS-assisted networks.

Owing to its efficiency in modeling long-range dependencies and spatial–temporal features, Mamba has increasingly been adopted in reinforcement learning applications. In [35], the authors proposed a hybrid trading framework that integrates Mamba state-space models, temporal convolutional networks, and attention mechanisms for feature extraction, combined with advanced DQN variants for reinforcement learning-based decision optimization, and demonstrated superior returns and risk-adjusted performance in high-frequency cryptocurrency trading. In [36], the authors proposed Mamba-DQN, which integrates the Mamba-SSM encoder and a full state sequence replay strategy into DQN to preserve temporal alignment, and achieved superior stability and sample efficiency over DQN, LSTM-DQN, and Transformer-DQN in dynamic environments.

## 3. Motivation and Contributions

IRS-assisted secure communications face several challenges in vehicular networks: the high mobility of vehicles causes rapidly varying channels; multiple VUs and potential eavesdroppers increase system complexity; and outdated CSI complicates the joint optimization of spectrum allocation, transmit power, and IRS phase shifts. Existing works typically assume simplified scenarios with static channels or a single eavesdropper, without considering dynamic spectrum sharing or multi-agent resource allocation. To the best of our knowledge, IRS-assisted interweave CIoV networks against malicious eavesdropping attacks have not been studied.

To address these challenges, we propose a multi-agent deep reinforcement learning (MADRL) framework under centralized training and decentralized execution (CTDE), capable of efficiently optimizing multi-user resource allocation and IRS phase shifts in dynamic environments.

The main contributions of this paper are listed in the following.

- We propose a novel IRS-assisted interweave CIoV network, accounting for outdated CSI, and formulate two optimization problems: maximizing the total minimum secrecy rate and minimizing the average maximum SOP. A heterogeneous MADRL approach is employed to solve these nonlinear and non-convex problems efficiently.
- We design a heterogeneous multi-agent Mamba RainbowDQN framework, where VUs handle transmit power and channel allocation as homogeneous agents, and the SBS acts as a heterogeneous agent to focus on IRS phase optimization, reducing computational burden and improving system flexibility.
- We integrate the Mamba module into the heterogeneous MADRL framework to enable the IRS to more effectively assist the interweave CIoV network in enhancing physical layer security. By capturing long-term temporal dependencies and high-dimensional state correlations, the proposed Mamba-enhanced MADRL allows agents to make more accurate and stable decisions under outdated CSI, and simulations show that the proposed framework outperforms baseline methods in system secrecy performance.

## 4. Paper Organization

The remainder of the paper is organized as follows. Section 5 introduces the IRS-assisted interweave CIoV network model under malicious eavesdropping attacks and formulates the corresponding optimization problem. Section 6 establishes the Markov Decision Process (MDP) framework and proposes the HMA-Mamba RainbowDQN algorithm to solve the optimization problem. Section 7 presents simulation results to evaluate the system performance via our proposed method as compared to benchmark schemes. Finally, Section 8 concludes this paper.

## 5. System Model and Problem Formulation

### 5.1. System Model

**Figure 1:**
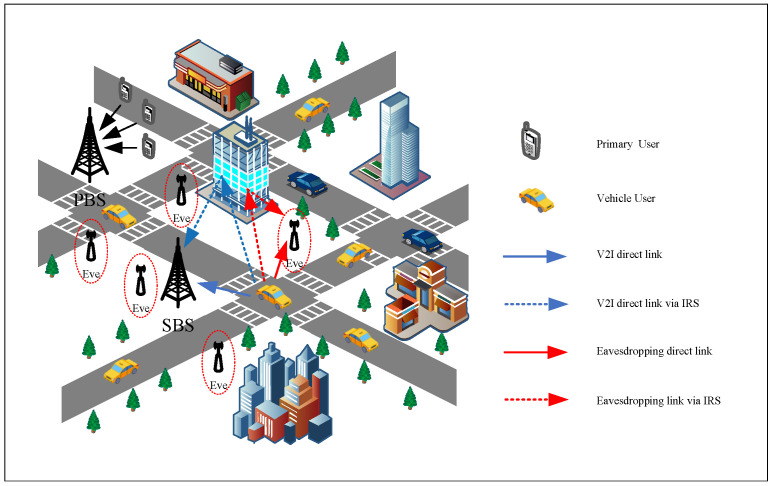
IRS-assisted interweave CIoV communication network under eavesdropping attacks.

In this paper, we consider an IRS-assisted interweave CIoV uplink network, as shown in Figure 1, consisting of a primary base station (PBS), *N* authorized primary users (PUs), a secondary base station (SBS) equipped with a single antenna [37,38], *I* vehicle users (VUs), *M* eavesdroppers (Eves), and an IRS with *L* reflecting elements controlled by the SBS. The PBS provides infrastructure support for PU communications but does not assist secondary systems. VUs independently detect PU activity through spectrum sensing, using conventional methods such as energy detection or cooperative sensing, and adopt an interweave transmission strategy, transmitting only when channels are sensed as idle.

The IRS-assisted secure communication framework we propose is governed by the geometric relationships among vehicles, the IRS, and the base station rather than the underlying road topology. To facilitate geometric modeling and illustrative clarity, a grid-based urban layout is adopted. Each VU randomly selects an initial direction and moves at a constant speed, turning at intersections according to predefined probabilities while maintaining connectivity with the SBS. Although vehicles in real urban scenarios frequently accelerate or decelerate, this simplification enables tractable mobility modeling and focuses the analysis on IRS optimization and secure V2I transmission, providing an indicative upper bound on system performance.

Each PU is allocated a dedicated orthogonal channel to avoid mutual interference so that the number of channels equals the number of PUs. The activity of each PU is modeled as a two-state Markov chain (idle/active), with details given in Appendix A.

### 5.2. Eavesdropping Model

Unlike traditional physical-layer security studies that often assume a single eavesdropper, in practical vehicular networks, malicious nodes may be distributed at critical locations such as intersections or toll stations. To capture this, we consider a multi-eavesdropper scenario where *M* eavesdroppers (M≥2) are randomly deployed within a circular region of radius 100 m centered at the SBS, with their coordinates denoted by $E=(x_{m},y_{m})|m=1,2,\ldots ,M$.

To rigorously and conservatively evaluate secrecy performance, we adopt a worst-case security assumption [39,40]. Specifically, each Eve is modeled as a powerful passive adversary capable of intercepting uplink transmissions from all VUs. The eavesdroppers act independently and do not share or aggregate intercepted signals. For analytical tractability and to ensure conservative results, each Eve is further assumed to ideally separate and decode the multi-user signals without intrinsic information loss (e.g., via optimal multi-user detection).

Under this assumption, the instantaneous secrecy rate of each VU is defined against the strongest eavesdropper, i.e., with respect to the maximum eavesdropping rate among all Eves. This does not imply cooperation, but rather reflects a worst-case perspective: since the system may be compromised once the most capable Eve succeeds, secrecy performance is conservatively evaluated against the strongest adversary. Moreover, for IRS-assisted transmission design, we assume that the SBS has perfect knowledge of the CSI of all Eves, which enables a robust assessment of system security under the most challenging conditions.

### 5.3. Communication Model

Let the coordinates of the SBS, PBS, and IRS be $\left(x_{\mathrm{SBS}},y_{\mathrm{SBS}}\right)$, $\left(x_{\mathrm{PBS}},y_{\mathrm{PBS}}\right)$, and $\left(x_{\mathrm{IRS}},y_{\mathrm{IRS}}\right)$, respectively. At the *t*-th time slot, the coordinates of the *i*-th VU and the *m*-th Eve are $\left(x_{i},y_{i}\right)$ and $\left(x_{m},y_{m}\right)$, respectively. The channel gain between the *i*-th VU and the SBS can be defined as      

(1) $$h_{i,S}=\sqrt{l_{0}\;\phi {\left(d_{i,S}\right)}^{-\alpha }}\;g_{i,S},$$

where $l_{0}$ denotes the path loss at a reference distance of 1 m, ϕ represents log-normal shadowing, $d_{i,S}$ is the distance between the *i*-th VU and the SBS, α is the path-loss exponent, and $g_{i,S}$ denotes the small-scale fading component, which follows a Rayleigh distribution.

Similarly, the channel gains between the *i*-th VU and the IRS, and between the IRS and the SBS, are defined as

(2) $$\begin{array}{cc} h_{i,R} & =\sqrt{l_{0}\;\phi {\left(d_{i,R}\right)}^{-\alpha }}\;e^{-j2\pi d_{i,R}/\lambda }\;a_{\mathrm{AoA}}, \end{array}$$

(3) $$\begin{array}{cc} h_{R,S} & =\sqrt{l_{0}\;\phi {\left(d_{R,S}\right)}^{-\alpha }}\;e^{j2\pi d_{R,S}/\lambda }\;a_{\mathrm{AoD}}, \end{array}$$

where $d_{i,R}$ and $d_{R,S}$ denote the distances between the *i*-th VU and the IRS, and between the IRS and the SBS, respectively; λ is the carrier wavelength. $a_{\mathrm{AoA}}$ and $a_{\mathrm{AoD}}$ are the array response vectors for the IRS incident and reflected links, given by

(4) $$\begin{array}{cc} a_{\mathrm{AoA}} & ={\left[1,e^{-j2\pi \frac{d}{\lambda }\sin\left(\theta_{\mathrm{AoA}}\right)},\ldots ,e^{-j2\pi \frac{d}{\lambda }\left(L-1\right)\sin\left(\theta_{\mathrm{AoA}}\right)}\right]}^{T}, \end{array}$$

(5) $$\begin{array}{cc} a_{\mathrm{AoD}} & ={\left[1,e^{-j2\pi \frac{d}{\lambda }\sin\left(\theta_{\mathrm{AoD}}\right)},\ldots ,e^{-j2\pi \frac{d}{\lambda }\left(L-1\right)\sin\left(\theta_{\mathrm{AoD}}\right)}\right]}^{T}. \end{array}$$

where $\theta_{\mathrm{AoA}}$ and $\theta_{\mathrm{AoD}}$ denote the angle of arrival and departure at the IRS, respectively, and *d* is the IRS element spacing. Similarly, the channel gains between the *m*-th Eve and the VU or IRS are denoted by $h_{i,m}$ and $h_{R,m}$, respectively.

The IRS phase shift matrix is defined as

(6) $$\Theta =\mathrm{diag}\left\{\phi_{1},\phi_{2},\ldots ,\phi_{L}\right\},\;\phi_{l}=e^{j\theta_{l}},\;\;\theta_{l}\in \left[0,2\pi \right),\;\;\forall l,$$

where $\phi_{l}$ denotes the reflection coefficient of the *l*-th IRS element. Here, we assume ideal full-reflection, i.e., all amplitudes $\beta_{l}=1$ [29]. The phase shifts are practically hardware-limited and thus discretized in our action space modeling.

The performance of IRS-assisted communication critically depends on accurate CSI. In this work, the CSI of VUs is obtained via pilot-based channel estimation [41]. In high-mobility V2I scenarios, the rapid movement of vehicles leads to outdated CSI [22,42], which complicates the IRS phase shift design [43]. Therefore, the impact of outdated CSI is explicitly considered for V2I links. By contrast, eavesdropping links are assumed quasi-static due to the near-SBS static deployment of eavesdroppers [44], and outdated CSI is not considered.

Let $T_{s}$ denote the CSI acquisition delay. The outdated CSI can be modeled as

(7) $$h\left(t+T_{s}\right)=\kappa \tilde{h}\left(t\right)+\sqrt{1-\kappa^{2}}\Delta h,$$

where $\tilde{h}\left(t\right)$ is the estimated channel, Δh is the independent estimation error, and

(8) $$\kappa =J_{0}\left(2\pi f_{D}T_{s}\right)$$

represents the channel correlation coefficient with $f_{D}=vf_{c}/c$ being the maximum Doppler shift. A larger κ indicates stronger correlation and more accurate CSI, while smaller κ reflects faster channel variation. The actual V2I rate depends on $h(t+T_{s})$ rather than the estimated CSI.

At time slot *t*, the Signal-to-Interference-plus-Noise Ratio (SINR) of the *i*-th VU on channel *n* is given by

(9) $$\gamma_{i}^{n}\left(t\right)=\frac{p_{i}|h_{i,S}+h_{R,S}^{H}\Theta h_{i,R}{|}^{2}}{\sum_{k\in I,k\neq i}\delta_{k}^{n}\;p_{k}|h_{k,S}+h_{R,S}^{H}\Theta h_{k,R}{|}^{2}+\sigma_{i}^{2}},$$

where $p_{i}$ denotes the transmit power of the *i*-th VU, $\sigma_{i}^{2}$ is the noise power, and $\delta_{k}^{n}\in \left\{0,1\right\}$ indicates whether the *k*-th VU accesses channel *n*. It should be emphasized that the interference term only accounts for VUs sharing channel *n* as indicated by $\delta_{k}^{n}$ since different channels are assumed orthogonal in cognitive radio systems. Thus, only co-channel users contribute to the interference of the *i*-th VU.

Accordingly, the achievable data rate is expressed as

(10) $$C_{i}^{n}\left(t\right)=b_{n}\left(t\right)W\log_{2}\left(1+\gamma_{i}^{n}\left(t\right)\right),$$

where *W* is the channel bandwidth, and $b_{n}\left(t\right)\in \left\{0,1\right\}$ represents the channel state $b_{n}\left(t\right)=1$ if the channel is idle, and $b_{n}\left(t\right)=0$ if occupied by PUs.

Similarly, the SINR experienced at the *m*-th Eve when eavesdropping on the *i*-th VU is given by

(11) $$\gamma_{m,i}^{n}\left(t\right)=\frac{p_{i}|h_{i,m}+h_{R,m}^{H}\Theta h_{i,R}{|}^{2}}{\sum_{k\in I,k\neq i}\delta_{k}^{n}\;p_{k}|h_{k,m}+h_{R,m}^{H}\Theta h_{k,R}{|}^{2}+\sigma_{m}^{2}},$$

and the corresponding eavesdropping rate is

(12) $$C_{m,i}^{n}\left(t\right)=b_{n}\left(t\right)W\log_{2}\left(1+\gamma_{m,i}^{n}\left(t\right)\right).$$

To evaluate secrecy performance rigorously, we adopt a worst-case assumption: each eavesdropper may intercept transmissions from all VUs, and the instantaneous secrecy rate of the *i*-th VU is defined using the maximum eavesdropping rate among all eavesdroppers:

(13) $$R_{i}^{\sec}\left(t\right)={\left[C_{i}^{n}\left(t\right)-\max_{m\in M}C_{m,i}^{n}\left(t\right)\right]}^{+},$$

where ${\left[z\right]}^{+}=\max\left(0,z\right)$. The total secrecy rate of the system at time slot *t* is

(14) $$R^{\sec}\left(t\right)=\sum_{i=1}^{I}R_{i}^{\sec}\left(t\right).$$

While $R^{\sec}\left(t\right)$ measures the instantaneous secrecy of the V2I link, the SOP [31] captures its robustness and is defined as

(15) $$P^{\mathrm{out}}\left(t\right)=\mathrm{Pr}\left\{R^{\sec}\left(t\right)<r_{\mathrm{th}}\right\},$$

where $r_{\mathrm{th}}$ is the target secrecy threshold.

### 5.4. Problem Formulation

In this work, we formulate two optimization problems to enhance the physical layer security of IRS-assisted V2I communications.

(1) *Secrecy Rate Maximization*

The first problem aims to maximize the total secrecy rate of the system by jointly optimizing the IRS phase shift matrix Θ, the transmit power vector $p=[p_{1},\ldots ,p_{I}]$, and the channel allocation matrix $\delta =[\delta_{i}^{n}]$:  

(16a) $$\begin{array}{cc} P_{1}:\; & \max_{\Theta ,p,\delta }\;R_{\mathrm{sum}}^{\sec}\left(t\right), \end{array}$$

(16b) $$\begin{array}{cc} s.t.\;\; & R_{i}^{\sec}\left(t\right)\geq R_{i}^{\left(\sec,\min\right)},\;\forall i\in I, \end{array}$$

(16c) $$\begin{array}{cc} \; & C_{i}^{n}\left(t\right)\geq C_{i}^{\min},\;\forall i\in I,\forall n\in N, \end{array}$$

(16d) $$\begin{array}{cc} \; & 0\leq \theta_{l}<2\pi ,\;\forall l\in L, \end{array}$$

(16e) $$\begin{array}{cc} \; & 0\leq p_{i}\leq p_{\max},\;\forall i\in I, \end{array}$$

(16f) $$\begin{array}{cc} \; & \delta_{i}^{n}\in \left\{0,1\right\},\;\forall i\in I,\forall n\in N, \end{array}$$

(16g) $$\begin{array}{cc} \; & \sum_{n=1}^{N}\delta_{i}^{n}\leq 1,\;\forall i\in I. \end{array}$$

Constraints C1–C2 guarantee the minimum secrecy and data rate requirements of each VU, C3 restricts the IRS phase shifts, C4 limits the transmit power, and C5–C6 ensure that each VU occupies at most one channel.

(2) *Secrecy Outage Probability Minimization*

(17a) $$\begin{array}{cc} P_{2}:\; & \min_{\Theta ,p,\delta }\;\max_{i\in I}P_{i}^{\mathrm{out}}\left(t\right), \end{array}$$

(17b) $$\begin{array}{cc} s.t.\;\; & C_{i}^{n}\left(t\right)\geq r_{\mathrm{th}},\;\forall i\in I,\forall n\in N, \end{array}$$

(17c) $$\begin{array}{cc} \; & 0\leq \theta_{l}<2\pi ,\;\forall l\in L, \end{array}$$

(17d) $$\begin{array}{cc} \; & 0\leq p_{i}\leq p_{\max},\;\forall i\in I, \end{array}$$

(17e) $$\begin{array}{cc} \; & \delta_{i}^{n}\in \left\{0,1\right\},\;\forall i\in I,\forall n\in N, \end{array}$$

(17f) $$\begin{array}{cc} \; & \sum_{n=1}^{N}\delta_{i}^{n}\leq 1,\;\forall i\in I. \end{array}$$

Constraint C1 ensures that the achievable rate of each VU on the allocated channel is no less than the secrecy threshold $r_{\mathrm{th}}$, while the remaining constraints are consistent with those in problem $P_{1}$.

The joint optimization is non-convex due to coupled variables. Conventional methods (e.g., AO and SDR) are complex, require precise CSI, and provide only suboptimal solutions. DRL can learn near-optimal policies in unknown environments, but single-agent MDPs face large state–action spaces and inefficiency. Hence, we adopt a MADRL framework for improved flexibility and efficiency.

## 6. Deep Reinforcement Learning for Resource Allocation

### 6.1. Multi-Agent DRL Framework

Most existing works focus on homogeneous MADRL, assuming identical state and action spaces for all agents. However, in IRS-assisted interweave CIoV networks, the power and channel allocation actions of VUs differ from the phase shift actions of IRS, making this assumption unsuitable. Hence, we model the problem as an MDP with I + 1 heterogeneous agents, where VUs and the SBS are treated as two types of agents. Each interacts with the environment in discrete time steps to optimize its policy. This HMA strategy decomposes the large mixed (continuous–discrete) action space into manageable sub-tasks, alleviating the “curse of dimensionality” and improving efficiency and convergence compared with single-agent methods.

### 6.2. Observation Space

At time slot *t*, the local observation state of the *i*-th VU agent is defined as

(18) $$s_{i}^{t}=\left\{O_{i}\left(t\right),h_{i}\left(t\right),h_{m,i}\left(t\right),G_{i}\left(t-1\right)\right\},$$

where $O_{i}\left(t\right)=\left\{O_{i}^{1}\left(t\right),\ldots ,O_{i}^{N}\left(t\right)\right\}$ denotes the spectrum sensing result set of the VU, and $O_{i}^{n}\left(t\right)\in \left\{0,1\right\}$ indicates the activity of the PU on the *n*-th channel (1: busy, 0: idle). $h_{i}\left(t\right)=\left\{h_{i,S},h_{i,R},h_{R,S}\right\}$ represents the CSI related to this VU, and $h_{m,i}\left(t\right)=\left\{h_{m,S},h_{m,R},h_{R,S}\right\}$ represents the CSI of all Eves eavesdropping on this VU. $G_{i}\left(t-1\right)$ denotes the co-channel interference from other VUs in the previous time slot.

The SBS agent is heterogeneous with respect to the VUs. Its local observation state is defined as

(19) $$s_{\mathrm{SBS}}^{t}=\left\{\Theta \left(t-1\right),\left\{h_{i}\left(t\right)|i\in I\right\},\left\{h_{m,i}\left(t\right)|i\in I\right\}\right\},$$

where Θ(t−1) is the IRS phase shift matrix from the previous slot, and $\left\{h_{i}\left(t\right)\right\}$ and $\left\{h_{m,i}\left(t\right)\right\}$ denote the CSI sets of all VUs and their corresponding Eves, respectively. To enable IRS optimization, VUs periodically report limited local information to the SBS, forming a lightweight exchange that remains feasible even in high-speed vehicular scenarios.

### 6.3. Action Space

In time slot *t*, each VU agent jointly decides on channel selection and power allocation. The transmit power is quantized into $A_{p}$ discrete levels, and one channel $c_{i}$ is selected from *N* available channels. Accordingly, the action space size is $|A_{\mathrm{VU}}|=NA_{p}$, given by

(20) $$A_{\mathrm{VU}}=\left\{a_{\left(c_{i},p_{j}\right)}|i=1,\ldots ,N;\;j=1,\ldots ,A_{p}\right\},$$

where $c_{i}$ denotes the *i*-th channel and $p_{j}$ the *j*-th power level.

For the SBS agent, the action in slot *t* is the phase shift variation ΔΘ(t), updated as

(21) Θ(t)=Θ(t−1)⊙ΔΘ(t),

where ⊙ denotes element-wise multiplication. Each ΔΘ(t) is selected from a subset of the discrete Fourier transform (DFT) basis vectors

(22) $$v\left(\psi \right)={\left[1,e^{j\pi \psi /L},\ldots ,e^{j\pi (L-1)\psi /L}\right]}^{⊤}.$$

Thus, the SBS action space is

(23) $$A_{\mathrm{SBS}}=\left\{v\left(\psi_{1}\right),v\left(\psi_{2}\right),\ldots ,v\left(\psi_{n}\right)\right\},$$

where $\psi_{k}$ (k=1,…,n) are predefined discrete values (e.g., rational fractions). This design fixes the action space size at $n\;\equiv \;|A_{\mathrm{SBS}}|$, independent of the number of IRS reflecting elements *L*, ensuring stable convergence even for large *L*.

### 6.4. Reward Design

To simultaneously maximize the total V2I secrecy rate and minimize the maximum SOP while strictly satisfying QoS constraints, the reward function at time slot *t* is defined as

(24) $$r_{t}=\mu_{1}\frac{R^{\sec}\left(t\right)}{R_{\max}^{\sec}}-\mu_{2}\log\left(1+P^{\mathrm{out}}\left(t\right)\right)-\mu_{3}\sum_{i=1}^{I}\left[\Phi_{i}^{\mathrm{QoS}}\left(t\right)+\Phi_{i}^{\sec}\left(t\right)\right],$$

where $\mu_{1},\mu_{2},\mu_{3}$ are weighting parameters to balance different objectives during training, and $R_{\max}^{\sec}$ denotes the theoretical maximum secrecy rate.

The QoS penalty for the *i*-th VU is defined as

(25) $$\Phi_{i}^{\mathrm{QoS}}\left(t\right)=\left\{\begin{array}{cc} {\left(\max(0,C_{i}^{\min}-C_{i}^{n}\left(t\right))\right)}^{2}, & 0<C_{i}^{n}\left(t\right)\\ \rho_{\mathrm{QoS}}, & \mathrm{otherwise} \end{array}\right.$$

where $\rho_{\mathrm{QoS}}>0$ is a constant to ensure sufficient punishment when the VU selects a PU-occupied channel, leading to a zero transmission rate.

The secrecy-rate penalty for the *i*-th VU is defined as

(26) $$\Phi_{i}^{\sec}\left(t\right)=\left\{\begin{array}{cc} {\left(\max(0,R_{i}^{\left(\sec,\min\right)}-R_{i}^{\sec}\left(t\right))\right)}^{2}, & 0<R_{i}^{\sec}\left(t\right)\\ \rho_{\sec}, & \mathrm{otherwise} \end{array}\right.$$

where $\rho_{\sec}>0$ ensures sufficient punishment when the secrecy rate falls below the minimum required threshold.

### 6.5. HMA-Mamba RainbowDQN Algorithm

As the baseline algorithm, Rainbow DQN integrates multiple enhancements to improve the learning efficiency and stability of traditional DQN [45]. Prioritized experience replay (PER) adjusts sampling probabilities based on the TD error $|\delta_{t}|$, defined as

(27) $$P_{\mathrm{sam},t}\propto {\left|\delta_{t}\right|}^{\omega },$$

enabling the model to focus on critical samples. Double DQN (DDQN) decouples action selection from evaluation, with the target value formulated as

(28) $$Q_{target}^{DDQN}=r_{t}+\gamma Q_{\theta^{-}}\left(s_{t+1},\arg\max_{a}Q_{\theta }\left(s_{t+1},a\right)\right),$$

which effectively mitigates Q-value overestimation. Dueling DQN separates the state value and advantage functions, computed as

(29) $$Q\left(s,a\right)=V\left(s\right)+\left(A\left(s,a\right)-\frac{1}{\left|A\right|}\sum_{a^{\prime }}A\left(s,a^{\prime }\right)\right),$$

providing a more accurate assessment of state importance. Multi-step Learning incorporates n-step returns, expressed as

(30) $$Q_{target}^{\left(n\right)}=\sum_{k=0}^{n-1}\gamma^{k}r_{t+k}+\gamma^{n}\max_{a}Q_{\theta^{-}}\left(s_{t+n},a\right),$$

to balance convergence speed and stability. NoisyNet introduces learnable noise into parameters, such as

(31) $$W=\mu^{W}+\sigma^{W}⊙\epsilon^{W},$$

achieving adaptive exploration. Finally, Distributional RL (C51) learns the full return distribution Z(s,a) instead of only its expectation, with the target distribution defined as

(32) $$TZ\left(s_{t},a_{t}\right)=r_{t}+\gamma Z\left(s_{t+1},a*\right),\;a*=\arg\max_{a}E\left[Z\left(s_{t+1},a\right)\right],$$

which captures uncertainty in value estimates and enhances policy robustness.

**Figure 2:**
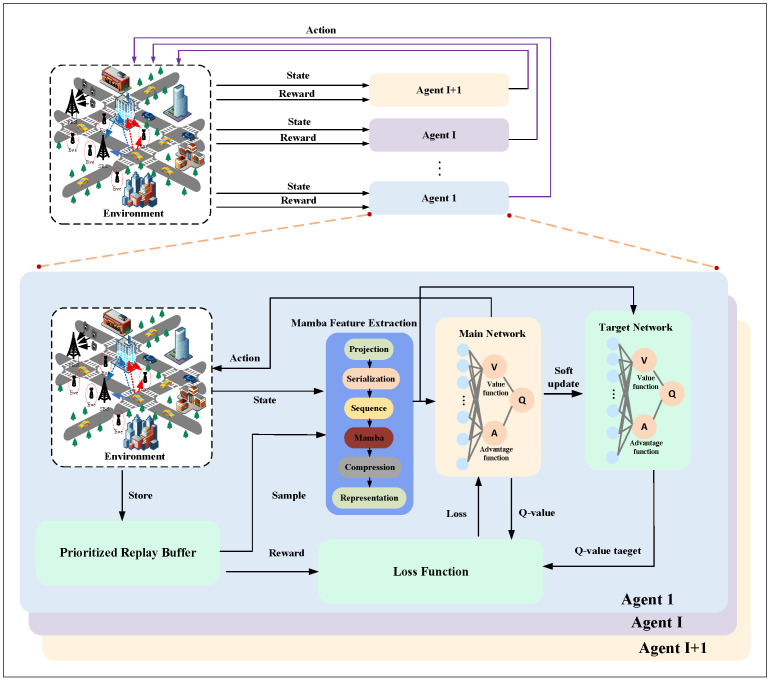
Representation of HMA-Mamba RainbowDQN framework.

In conventional DQN, feature extraction primarily relies on MLPs or CNNs. However, MLPs struggle to capture temporal dependencies, while CNNs, although proficient in local pattern recognition, are limited in representing high-dimensional abstract states, particularly those involving historical trajectory information. This often leads to insufficient state representation and training instability. In contrast, Mamba exhibits strong sequential modeling capability, enabling it to automatically capture dynamic relationships among states with high computational efficiency [46]. Leveraging this advantage, this work incorporates Mamba into the Rainbow DQN framework and proposes the heterogeneous multi-agent Mamba RainbowDQN method as shown in Figure 2.

It should be noted that the original Rainbow incorporates C51 distributional learning; however, in multi-agent CIoV scenarios, this approach may induce environmental non-stationarity and suboptimal solutions. Therefore, in this work, C51 is omitted, and the centralized training with decentralized execution (CTDE) paradigm is adopted to ensure training stability and execution efficiency. Under CTDE, agents learn coordinated policies guided by a shared system-level reward during centralized training, while executing their actions independently based on local observations during deployment.

**Figure 3:**
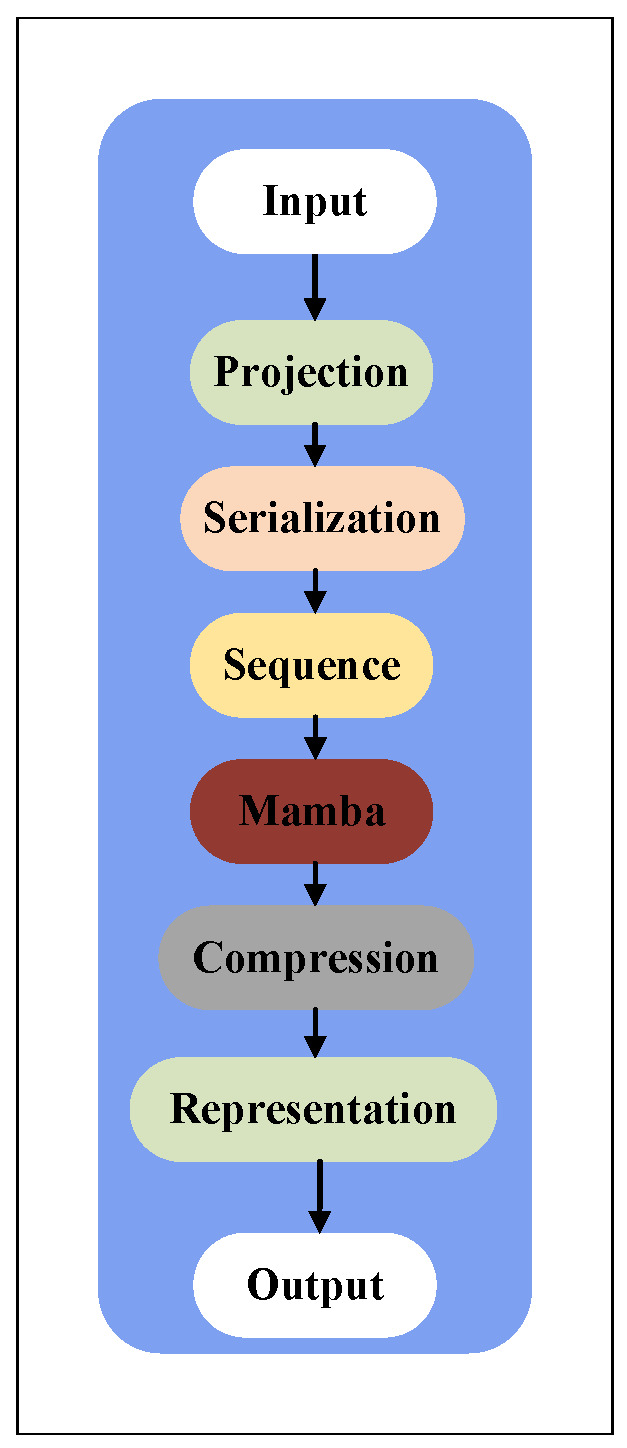
Mamba State Feature Extraction Module.

As shown in Figure 3, the Mamba state feature extraction pipeline projects the raw environment state into a hidden space, serializes it into a sequential structure, and applies state-space modeling before compression into a high-dimensional feature.

Formally, the environment state vector $s\in R^{d_{\mathrm{state}}}$ is first projected as

(33) $$s_{\mathrm{proj}}=W_{\mathrm{in}}s+b_{\mathrm{in}},$$

where $W_{\mathrm{in}}\in R^{d_{\mathrm{hidden}}\times d_{\mathrm{state}}}$ and $b_{\mathrm{in}}\in R^{d_{\mathrm{hidden}}}$.

To fit the sequential modeling structure of Mamba, $s_{\mathrm{proj}}$ is reshaped into a sequence:

(34) $$s_{\mathrm{seq}}=\mathrm{unsqueeze}\left(s_{\mathrm{proj}},\;\dim=1\right),$$

resulting in an input of shape $\left(1,d_{\mathrm{hidden}}\right)$. Despite the sequence length being one, Mamba is designed to capture temporal dependencies and perform convolutional expansion along the feature dimension, allowing the extraction of rich, abstract features:

(35) $$s_{\mathrm{mamba}}=\mathrm{Mamba}\left(s_{\mathrm{seq}}\right).$$

Finally, the output sequence is compressed to yield a fixed-length feature vector

(36) $$s_{\mathrm{mamba}\_\mathrm{flat}}=\mathrm{squeeze}\left(s_{\mathrm{mamba}},\;\dim=1\right),\;s_{\mathrm{mamba}\_\mathrm{flat}}\in R^{d_{\mathrm{hidden}}},$$

which is then fed into the Dueling architecture.

Next, the Dueling architecture decomposes this representation into state-value and advantage functions. The state-value function is

(37) $$V\left(s_{t}|w,\beta \right)=W_{V}s_{\mathrm{mamba}\_\mathrm{flat}}+b_{V},$$

where $W_{V}\in R^{1\times d_{\mathrm{hidden}}}$, $b_{V}\in R$. The advantage function is

(38) $$A\left(s_{t},a_{t}|w,\alpha \right)=W_{A}s_{\mathrm{mamba}\_\mathrm{flat}}+b_{A},$$

where $W_{A}\in R^{\left|A\right|\times d_{\mathrm{hidden}}}$, $b_{A}\in R^{\left|A\right|}$. To eliminate action-scale bias, the advantage function is normalized to zero mean. The final Q-value is then expressed as

(39) $$Q\left(s_{t},a_{t}|w,\alpha ,\beta \right)=V\left(s_{t}|w,\beta \right)+\left(A\left(s_{t},a_{t}|w,\alpha \right)-\frac{1}{\left|A\right|}\sum_{a^{\prime }\in A}A\left(s_{t},a^{\prime }|w,\alpha \right)\right).$$

In Mamba Rainbow, the Q-value target is defined as

(40) $$y_{t}^{\mathrm{MambaRainbow}}=\sum_{k=0}^{n-1}\gamma^{k}r_{t+k}+\gamma^{n}Q\left(s_{t+n},\arg\max_{a}Q\left(s_{t+n},a;w,\alpha ,\beta \right);w^{-},\alpha^{-},\beta^{-}\right),$$

where $w^{-},\alpha^{-},\beta^{-}$ denote the parameters of the target network, and the sampling priority is determined by $|\delta_{t}{|}^{\alpha }$.

Based on this target, the loss function with PER is

(41) $$L\left(w,\alpha ,\beta \right)=E_{(s,a,r,s^{\prime })∼D}\left[w_{i}\cdot {\left(y_{t}^{\mathrm{MambaRainbow}}-Q\left(s_{t},a_{t}|w,\alpha ,\beta \right)\right)}^{2}\right],$$

where each transition *i* is sampled with probability

(42) $$P\left(i\right)=\frac{|\delta_{i}{|}^{\alpha }}{\sum_{j}{\left|\delta_{j}\right|}^{\alpha }},\;\delta_{i}=y_{t}^{\mathrm{MambaRainbow}}-Q\left(s_{t},a_{t}\right)$$

and the importance-sampling weight $w_{i}={\left(\frac{1}{N}\frac{1}{P(i)}\right)}^{\beta }$ corrects the bias introduced by prioritized sampling.

The target network parameters are updated via soft synchronization:

(43) $$\left\{\begin{array}{c} w^{-}\leftarrow \tau w+\left(1-\tau \right)w^{-},\\ \alpha^{-}\leftarrow \tau \alpha +\left(1-\tau \right)\alpha^{-},\\ \beta^{-}\leftarrow \tau \beta +\left(1-\tau \right)\beta^{-}. \end{array}\right.$$

The training process of the proposed HMA-Mamba RainbowDQN method is detailed in Algorithm 1.
**Algorithm 1** Proposed HMA-Mamba RainbowDQN method for IRS-assisted interweave CIoV network.**Input:** PU activity status, V2I link channel gain, CSI of VUs and Eve, co-channel interference from previous time slot, IRS phase shift matrix**Output:** Joint action $a_{t}$ of all agents, including VUs agents’ channel and power selection and SBS agent’s IRS phase adjustment      **Initialization:** Online network parameters Q(·) and target network parameters $Q^{-}\left(\cdot \right)$, prioritized experience replay buffer D, initial state of Mamba feature extractor $h_{0}=0$1:**for** each episode **do**2:    Initialize VUs positions and velocities, obtain initial environment state3:    **for** each time step *t* **do**4:        **for** each agent u=1,2,…,I+1 **do**5:           Obtain local observation $s_{t}^{u}$6:           Extract temporal features via Mamba: $f_{t}^{u}=\mathrm{Mamba}\left(s_{t}^{u};h_{t-1}\right)$7:           Select action from NoisyNet output: $a_{t}^{u}=\arg\max_{a}Q\left(f_{t}^{u},a;\theta_{\mathrm{noisy}}\right)$8:        **end for**9:        Execute joint action $a_{t}$, obtain reward $r_{t}$10:        **for** each agent u=1,2,…,I+1 **do**11:           Observe next state $s_{t+1}^{u}$12:           Store single-step experience $\left(s_{t}^{u},a_{t}^{u},r_{t},s_{t+1}^{u}\right)$ into buffer D13:        **end for**14:    **end for**15:    **for** each agent u=1,2,…,I+1 **do**16:        Sample a batch from D via prioritized sampling17:        Construct n-step returns and compute target $y_{t}^{\mathrm{MambaRainbow}}$ by Equation (40)18:        Compute gradients and update network parameters w,α,β by Equation (41)19:        Update the priorities of the sampled experiences based on TD-error20:        Soft-update target network parameters $w^{-},\alpha^{-},\beta^{-}$ by Equation (43)21:    **end for**22:**end for**

### 6.6. Computational Complexity Analysis

The computational complexity of the proposed HMA-Mamba RainbowDQN algorithm is primarily determined by the forward and backward propagation of the neural networks of all agents, with the linear scanning mechanism of the Mamba module as the core. For a single agent, the forward pass complexity is $T_{\mathrm{main}}=\sum_{z=0}^{Z-1}f_{z}f_{z+1}+\Phi \left(d_{\mathrm{state}}d_{\mathrm{hidden}}+d_{\mathrm{hidden}}^{2}\right)$, where *Z* is the number of fully connected layers (including the value and advantage streams in the Dueling architecture), $f_{z}$ is the number of neurons in the *z*-th layer, Φ is the number of stacked Mamba modules, and $d_{\mathrm{state}}$ and $d_{\mathrm{hidden}}$ are the input and hidden dimensions of the Mamba module. Let $N_{\mathrm{agents}}=I+1$ denote the total number of agents (including *I* VUs and one SBS). During training, which involves backpropagation, experience replay, and policy updates, the complexity scales linearly with the number of agents and is further multiplied by the number of episodes and steps, giving a total training complexity of $O\left(N_{\mathrm{episodes}}N_{\mathrm{steps}}N_{\mathrm{agents}}\left(2\sum_{z=0}^{Z-1}f_{z}f_{z+1}+\Phi \left(d_{\mathrm{state}}d_{\mathrm{hidden}}+d_{\mathrm{hidden}}^{2}\right)\right)\right)$. In contrast, during real-time execution, each agent only performs forward inference with per-step complexity $O(\sum_{z=0}^{Z-1}f_{z}f_{z+1}+\Phi \left(d_{\mathrm{state}}d_{\mathrm{hidden}}+d_{\mathrm{hidden}}^{2}\right))$, which scales linearly with the number of agents and is significantly lower than training, ensuring feasible online implementation while maintaining high system performance.

## 7. Simulation Results

In this section, simulations are conducted to evaluate the physical layer security performance of the proposed HMA-Mamba RainbowDQN algorithm under malicious eavesdropping attacks, with relevant parameters listed in Table 1. The SBS is located at (250, 150), the PBS at (100, 300), and the IRS is deployed at (200, 375). Each VU is equipped with a single antenna and randomly distributed along the lanes. PUs are randomly located within a circular area of 100 m radius centered at the PBS, while malicious Eves are randomly distributed within a circular area of 100 m radius centered at the SBS.

Experiments are conducted in Python 3.10 using PyTorch 2.3.1 on an NVIDIA GeForce RTX 4060 GPU (Nvidia Corporation, Santa Clara, CA, USA). The HMA-Mamba RainbowDQN framework maps environment states to a hidden dimension of $d_{\mathrm{hidden}}=256$ and uses the Mamba feature extractor with state dimension $d_{\mathrm{state}}=16$. The output Q-values are generated by a noisy linear layer. The target network is updated via soft and periodic hard updates, and experience replay uses a buffer of 100,000 with prioritized sampling. Exploration is performed using parameterized Gaussian Noisy Nets. Key training parameters are summarized in Table 2.

To evaluate the effectiveness of the proposed method, it is compared with the following benchmark schemes:

(1) HMA-RainbowDQN: a heterogeneous multi-agent RL algorithm that excludes the Mamba module, serving to isolate and highlight the contribution of Mamba-based feature extraction.
(2) HMA-D3QN and HMA-DQN: representative conventional DRL algorithms widely adopted in multi-agent resource allocation, used to demonstrate the relative advantages of the proposed framework over mainstream MADRL approaches.
(3) Random IRS Random RA: a baseline without intelligent optimization, where both IRS phase shifts and resource allocation are randomly determined.

**Figure 4:**
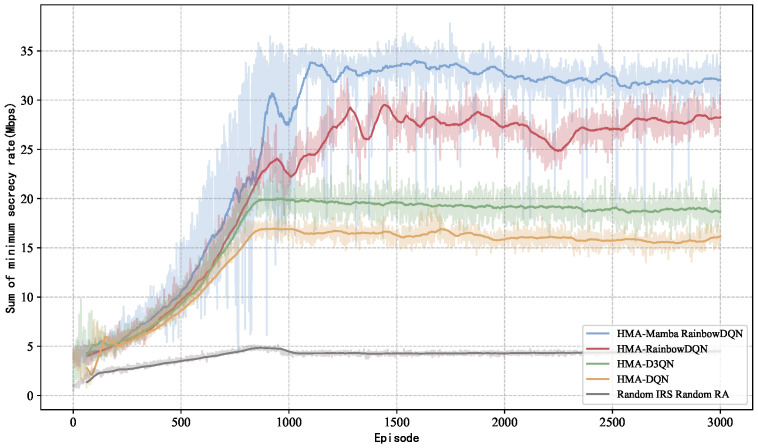
Minimum total secrecy rate versus the number of training episodes.

**Figure 5:**
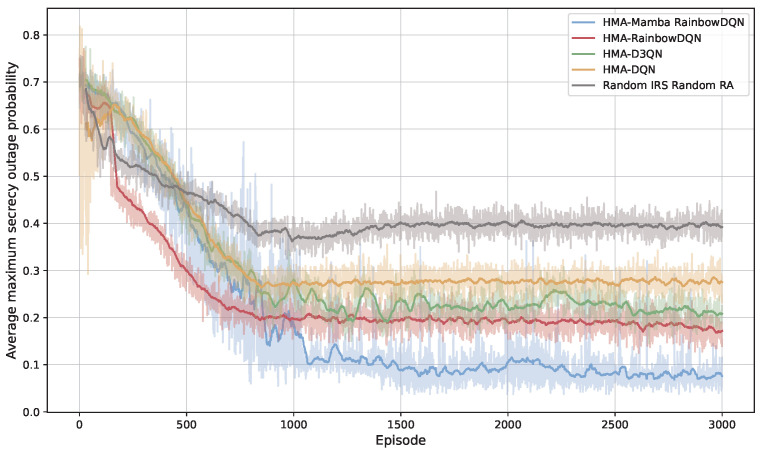
Average maximum SOP versus the number of training episodes.

Figure 4 and Figure 5 illustrate the per-episode minimum total secrecy rate and average maximum SOP during training for different algorithms. As shown in Figure 4, the random scheme consistently achieves the lowest secrecy rate, while the secrecy rate of other methods generally increase over training, with notable performance differences. Both HMA-D3QN and HMA-DQN converge to relatively low levels within the same number of training episodes, whereas the proposed HMA-Mamba RainbowDQN achieves the highest secrecy rate. Once training stabilizes, the proposed method demonstrates a 13.29% secrecy rate improvement over HMA-RainbowDQN. Figure 5 indicates that higher average minimum secrecy rate correspond to lower average maximum SOP, confirming that the proposed method also effectively minimizes system secrecy outages.

**Figure 6:**
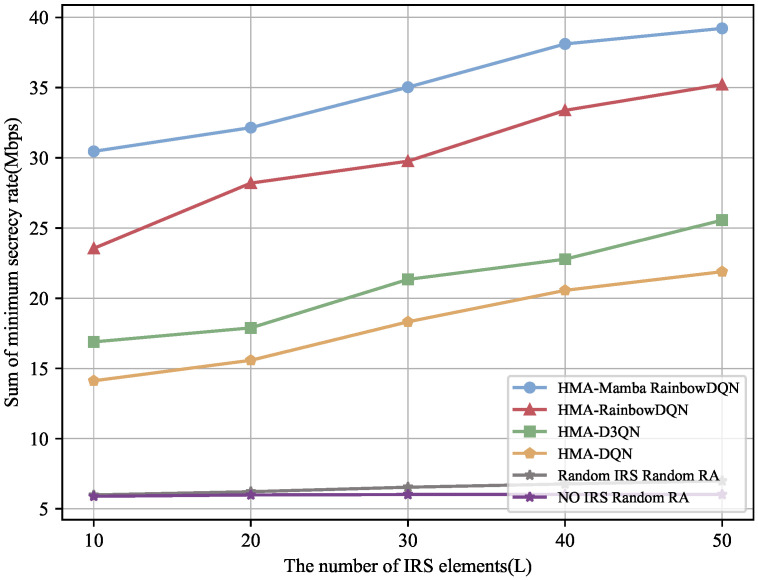
Minimum total secrecy rate versus the number of IRS elements.

**Figure 7:**
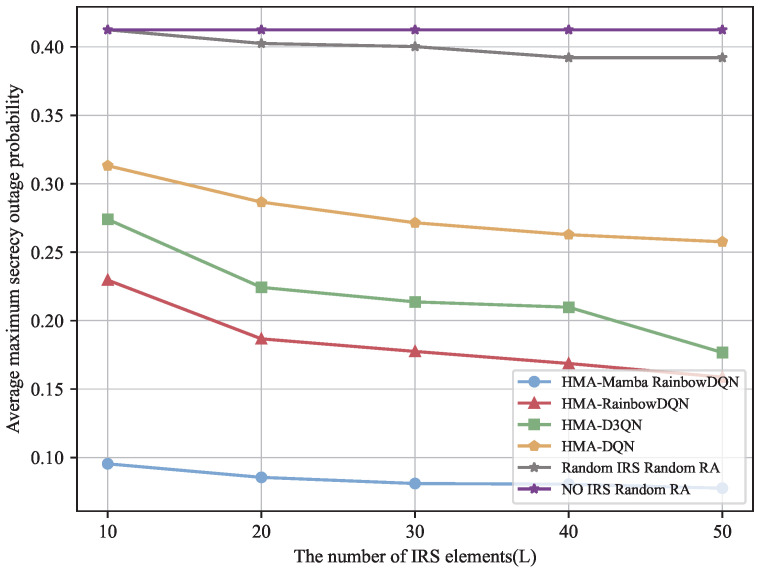
Average maximum SOP versus the number of IRS elements.

Figure 6 and Figure 7 examine the impact of the number of IRS reflecting elements on the minimum total secrecy rate and the average maximum SOP of the V2I link. To highlight the role of the IRS, a baseline scenario without IRS assistance and with randomly allocated VU resources is included for comparison. The results indicate that all IRS-based methods benefit from an increased number of reflecting elements, with the proposed HMA-Mamba RainbowDQN method showing the most significant improvement. This is because additional reflecting elements provide greater degrees of freedom to enhance the signal strength of legitimate channels while suppressing eavesdropping channels, thereby enlarging the difference between the two and improving physical layer security. In contrast, the randomly configured IRS scheme only slightly outperforms the no-IRS case, whose transmission rate remains nearly unchanged as the number of IRS reflecting elements increases, highlighting the importance of proper IRS deployment. Nevertheless, the selection of an appropriate number of reflecting elements is critical: too few elements may fail to satisfy the minimum communication rate requirements for all vehicles, whereas increasing the number can enhance security performance but may also introduce inter-user interference and higher computational complexity.

**Figure 8:**
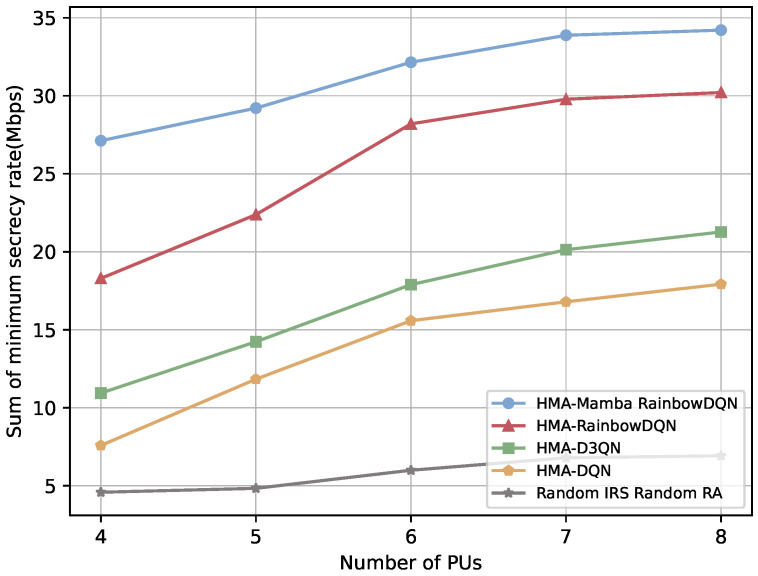
Minimum total secrecy rate versus the number of PUs.

**Figure 9:**
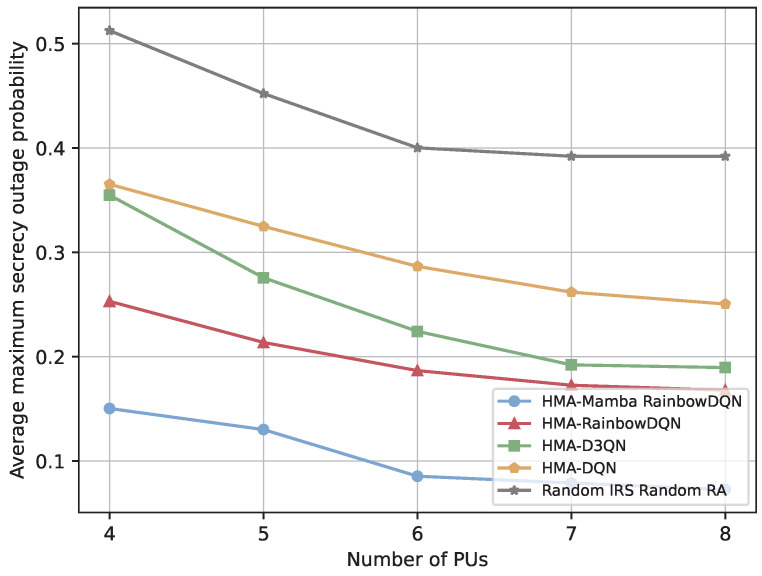
Average maximum SOP versus the number of PUs.

Figure 8 and Figure 9 illustrate the impact of the number of PUs on the secrecy performance of the V2I link. As the number of PUs increases, all schemes achieve higher secrecy rate and lower SOP. This improvement is primarily due to the increased availability of spectrum: more PUs provide additional orthogonal channels, enabling vehicles to strategically select channels with the lowest eavesdropping risk. Under limited channel resources (number of channels < number of VUs), secrecy performance is severely constrained, as VUs are forced to use high-risk channels. When channel availability becomes sufficient, the rate of secrecy improvement slows, indicating a diminishing marginal effect of additional spectrum on reducing eavesdropping risk. Notably, the proposed HMA-Mamba RainbowDQN consistently outperforms all benchmark algorithms, demonstrating its efficiency in identifying and selecting the safest channels in dynamic spectrum environments and achieving secure spectrum allocation even under persistent external eavesdropping threats.

**Figure 10:**
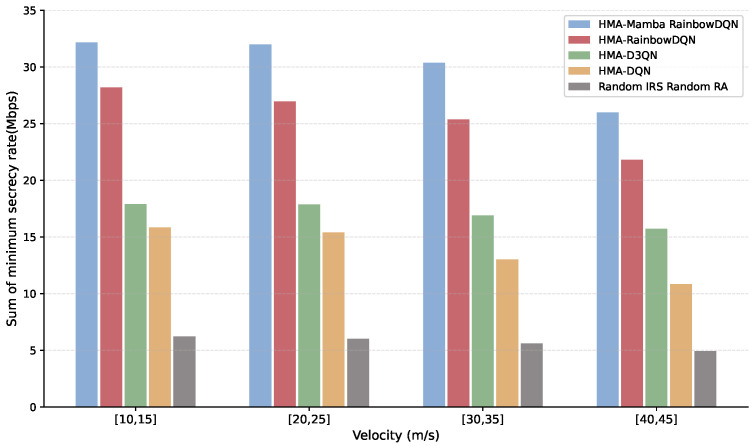
Minimum total secrecy rate versus VUs velocity.

**Figure 11:**
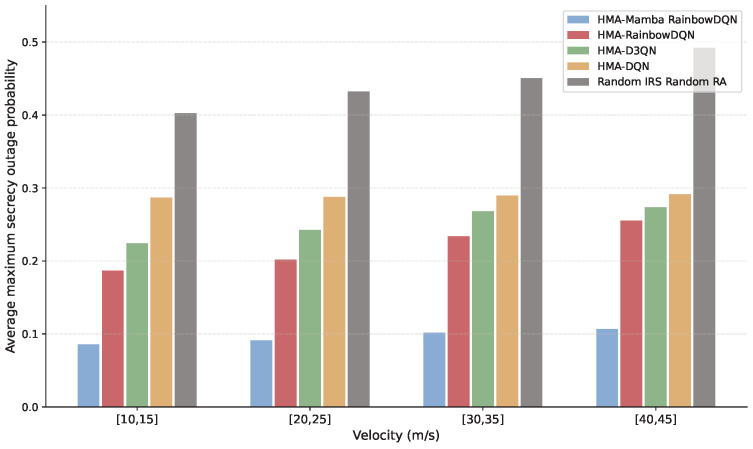
Average maximum SOP versus VUs velocity.

Figure 10 and Figure 11 illustrate the impact of VU mobility on the secrecy rate and SOP of the V2I link. As vehicle speed increases, the secrecy rate decreases while the SOP rises. This is primarily due to higher speeds increasing coverage distance and path loss, as well as more rapid changes in the environment, which degrade the timeliness of CSI. Additionally, increased speed amplifies Doppler shifts $f_{c}$, causing fast channel variations and reducing channel estimation accuracy. Specifically, as speed increases from [10, 15] m/s to [40, 45] m/s, the proposed HMA-Mamba RainbowDQN and the HMA-RainbowDQN, HMA-D3QN, and HMA-DQN algorithms experience approximately 10–30% reduction in secrecy rate and 20–40% increase in SOP. Nevertheless, the proposed method consistently outperforms the benchmarks across all speeds, demonstrating its robustness to mobility-induced environmental changes and its ability to maintain stable physical layer security performance.

**Figure 12:**
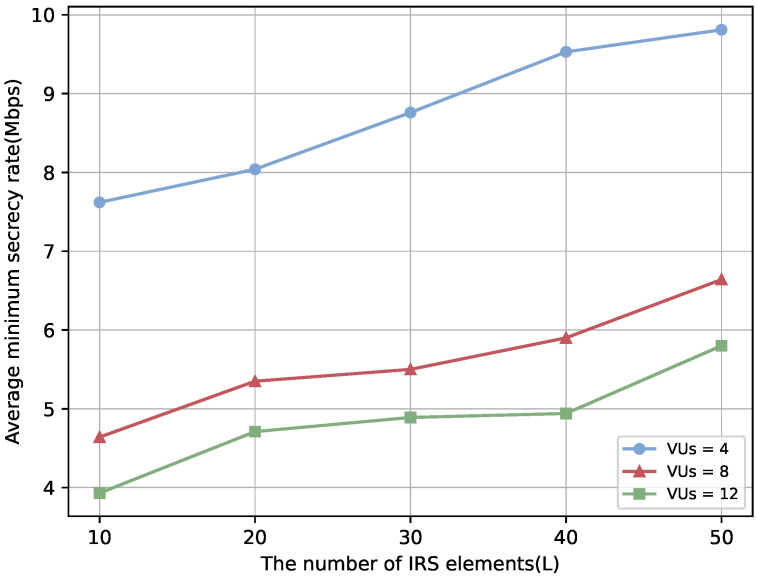
Average minimum secrecy rate over the number of IRS elements with different number of VUs.

**Figure 13:**
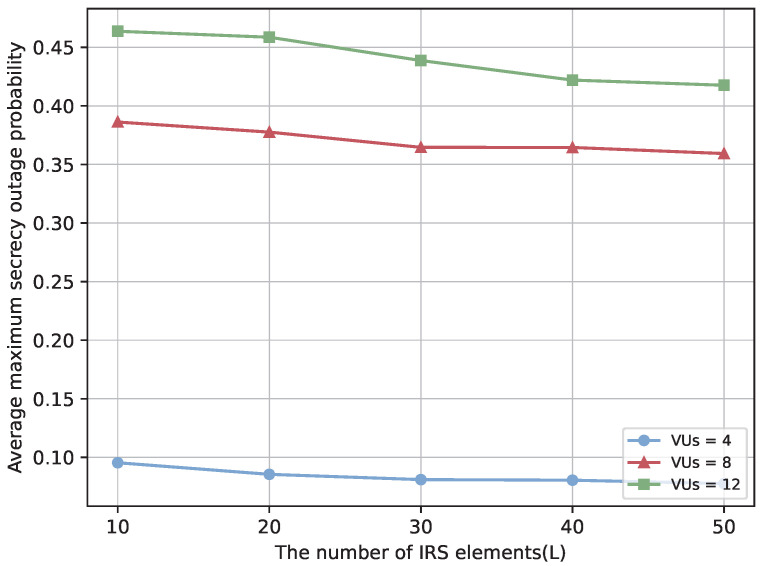
Average SOP over the number of IRS elements with different number of VUs.

To enhance the comprehensiveness of the study, we further analyze the impact of increasing the number of VUs on system performance. Figure 12 and Figure 13 show that the secrecy performance of each user improves with the number of IRS elements. However, as the number of VUs grows, the average minimum secrecy rate per VU decreases, while the average SOP increases. This is primarily due to the limited spectrum, power, and IRS reflection resources being shared among more users, reducing the effective channel gain per VU. Meanwhile, increased inter-user interference and channel correlation make it difficult for the IRS phase configuration to accommodate all users, weakening its capability to enhance legitimate links and suppress eavesdropping. Furthermore, under the worst-case criterion, the secrecy performance of each VU is determined by the Eve with the highest achievable rate. As the number of VUs increases, some users are more likely to face excessively strong eavesdropping channels, significantly lowering their secrecy rates and degrading overall system performance. These observations indicate that while IRS deployment can improve system performance, the challenges introduced by a larger number of VUs must be carefully considered.

Table 3 presents the average training runtime of different algorithms under the malicious eavesdropping scenario (evaluated over 100 episodes). As expected, the proposed HMA-Mamba RainbowDQN requires significantly longer training time than the benchmark schemes. Compared with DQN and D3QN, RainbowDQN inherently incurs additional computational overhead due to the integration of modules such as NoisyNet and prioritized experience replay. On top of this, the incorporation of the Mamba architecture introduces further complexity from the state space model (SSM) and selective scanning mechanism, thereby prolonging the overall training process. Nevertheless, as demonstrated by the superior secrecy performance reported earlier, this additional training overhead can be regarded as a reasonable trade-off for enhancing physical layer security.

**Figure 14:**
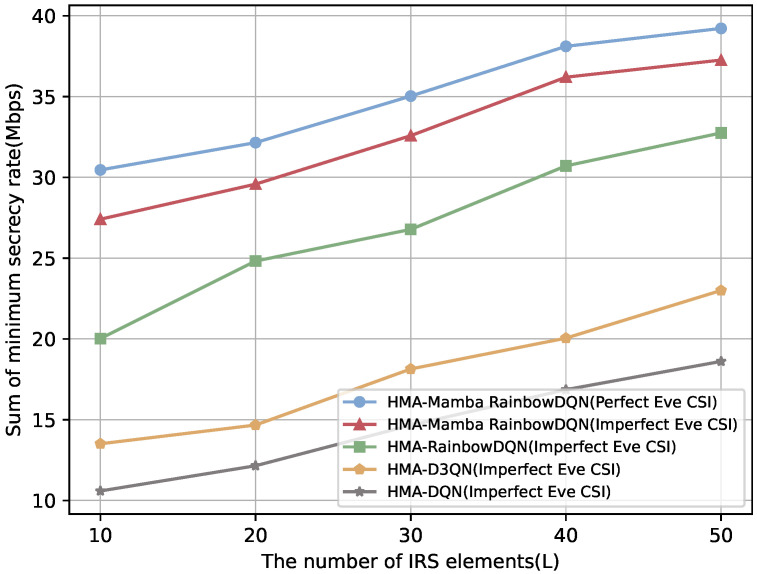
Comparison of secrecy rate versus the number of IRS elements under imperfect Eve CSI.

**Figure 15:**
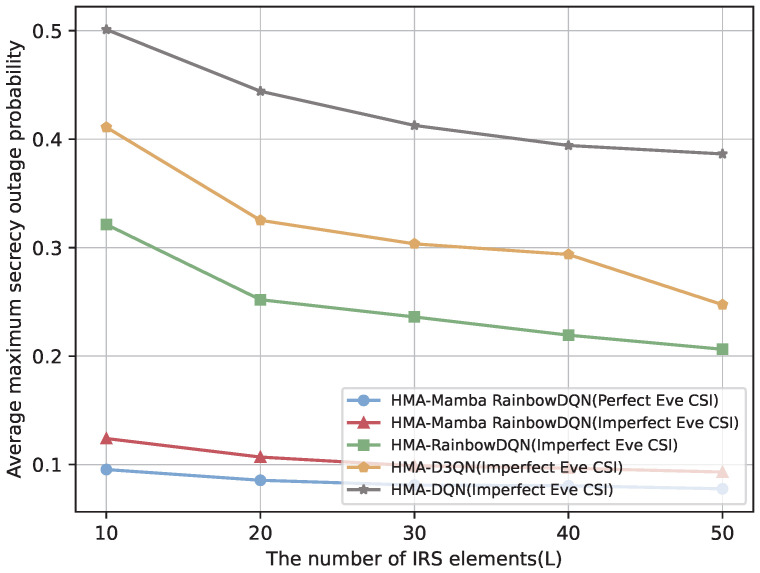
Comparison of SOP versus the number of IRS elements under imperfect Eve CSI.

It is worth noting that the preceding experiments were conducted under the baseline assumption of perfect CSI at the eavesdroppers, a common simplification for analytical tractability. To assess the robustness of the proposed scheme under more practical conditions, we further consider the case of imperfect Eve CSI. In this setting, Eve’s channels are modeled using the outdated CSI assumption together with a norm-bounded error model as in [47], with the detailed formulation provided in Appendix B. As illustrated in Figure 14 and Figure 15, all schemes experience a degradation in secrecy rate and an increase in SOP when Eve’s CSI is imperfect. Nevertheless, the proposed HMA-Mamba RainbowDQN consistently achieves the highest secrecy rate and the lowest SOP among the benchmarks, demonstrating its superiority and robustness against CSI uncertainty at the eavesdroppers.

## 8. Conclusions

This work investigates enhancing the physical layer security of interweave CIoV network under malicious eavesdropping using IRS. To maximize the total minimum secrecy rate and minimize the average maximum SOP, a non-convex joint optimization problem of IRS phase shifts, VU transmit power, and channel allocation is formulated. To address this problem, a HMA-Mamba RainbowDQN based resource allocation method is proposed, where VUs and the SBS act as heterogeneous agents interacting independently with the environment. Simulation results demonstrate that the proposed method significantly outperforms baseline schemes, achieving higher secrecy rate while effectively reducing secrecy outage risk; compared with the basic HMA-RainbowDQN, the secrecy rate increases by 13.29% and the SOP decreases by 54.2%. The design fully accounts for imperfect CSI, random PUs spectrum occupancy, and the stochastic positions and velocities of VUs, employing a worst-case criterion to counter independent non-cooperative Eves. These results validate the robustness and reliability of the proposed method in dynamic vehicular networks. The HMA-Mamba RainbowDQN demonstrates that integrating IRS with DRL can substantially enhance physical layer security in dynamic vehicular networks. The proposed method can be extended to broader dynamic scenarios, such as UAV networks or smart city IoT systems. For future research, we plan to extend the framework to multi-antenna (MISO/MIMO) BS scenarios and investigate highly mobile eavesdroppers with time-varying channels, as well as the deployment of multiple IRSs to further enhance the security and reliability of communications in large-scale or high-density vehicular networks.

## Figures and Tables

**Table 1:** Simulation parameters.

Parameter	Value
Number of VUs (*I*)	4
Number of PUs (*Q*)	6
Number of IRS elements (*L*)	18
Carrier frequency	2 GHz
Bandwidth	1 MHz
V2I transmit power ($p_{i}$)	[0, 23] dBm
BS and vehicles antenna gains	8, 3 dBi
BS and vehicles receiver noise gains	5, 11 dBi
Number of discrete power levels	9
Vehicle speed range	[10, 15] m/s
SBS antenna height	25 m
VUs antenna height	1.5 m
IRS height	25 m
Noise power	−114 dBm
V2I link path loss model	$128.1+37.6\log_{10}\left(d\right)$

**Table 2:** Neural network parameters.

Parameter	Value
Number of episodes	3000
Number of iterations per episode	100
Optimizer	Adam
Learning rate (μ)	0.001
Discount factor (γ)	0.99
Mamba internal activation	SiLU
Prioritized experience replay buffer size *D*	100,000
Target network soft update	0.005
Prioritization exponent (ω)	0.5
Prioritization type	proportional
Multi-step returns (*n*)	3
Exploration (ε)	0.0
Noisy Nets ($\sigma_{0}$)	0.5
SSM state dimension ($d_{\mathrm{state}}$)	16
Network hidden dimension ($d_{\mathrm{hidden}}$)	256
Expansion factor (*E*)	2

**Table 3:** Comparison of running time.

Algorithm	Time (s)
HMA-Mamba RainbowDQN	60.06
HMA-RainbowDQN	45.69
HMA-D3QN	24.67
HMA-DQN	21.45

**Figure A1:**
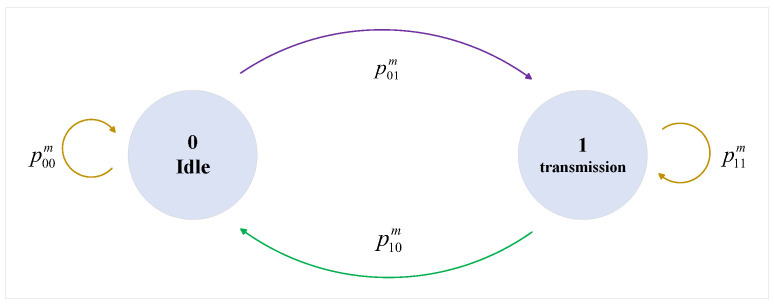
Two-state Markov model of PU activity.

## Data Availability

The original contributions presented in this study are included in the article. Further inquiries can be directed to the corresponding author.

## Appendix A

The activity of each PU is characterized by a two-state Markov chain as illustrated in Figure A1. The transition probability matrix for the *n*-th PU is given by

(A1) $$P_{n}=\left[\begin{array}{cc} p_{00} & p_{01}\\ p_{10} & p_{11} \end{array}\right],$$

where $p_{01}$ denotes the probability of transitioning from the idle state to the transmission state, and $p_{10}$ denotes the probability of transitioning from the transmission state to the idle state.

## Appendix B

Following [47], the imperfect CSI of each Eve is modeled by a norm-bounded error model. Let ${\hat{h}}_{i,m}$ and ${\hat{h}}_{R,m}$ denote the estimated direct and IRS to Eve channels, respectively. The actual channels are expressed as

(A2) $$h_{i,m}={\hat{h}}_{i,m}+{\Delta h}_{i,m},\;{∥{\Delta h}_{i,m}∥}^{2}\leq {\left(\varepsilon_{i,m}\right)}^{2},$$

(A3) $$h_{R,m}={\hat{h}}_{R,m}+{\Delta h}_{R,m},\;{∥{\Delta h}_{R,m}∥}^{2}\leq {\left(\varepsilon_{r,m}\right)}^{2},$$

where $\Delta h_{i,m}$ and $\Delta h_{R,m}$ denote bounded estimation errors.
